# Unusual Manifestations of Acute Cytomegalovirus Infection in Solid Organ Transplant Hosts: A Report of Two Cases

**DOI:** 10.1155/2017/4916973

**Published:** 2017-09-11

**Authors:** Alfredo Mena Lora, Justin Khine, Nadia Khosrodad, Vijay Yeldandi

**Affiliations:** ^1^Division of Infectious Diseases, Department of Medicine, University of Illinois at Chicago, Chicago, IL, USA; ^2^School of Medicine, Ross University, Miramar, FL, USA

## Abstract

Cytomegalovirus (CMV) infection is a common cause of morbidity and mortality in immunocompromised hosts. Tissue-invasive CMV disease causing ulcerative skin disease or esophageal necrosis is rare. We herein describe two cases: a 47-year-old renal and pancreas transplant recipient who presented with skin ulcerations on his elbow and a 50-year-old renal transplant recipient who presented with acute esophageal necrosis. In both, tissue biopsy revealed CMV inclusion bodies by immunohistochemical staining of infected endothelial and mucosal cells. Ganciclovir was given to both cases and full remission occurred. Due to the varying presentations of acute CMV infection in immunosuppressed hosts, high suspicion and early tissue biopsy are vital for proper diagnosis and treatment when any suspicious cutaneous or mucosal manifestations are present.

## 1. Introduction

Cytomegalovirus (CMV) is a Herpesviridae with high seroprevalence, estimated between 30 and 97% [1–3]. Primary infection in an immunocompetent host can be asymptomatic or only self-limiting disease [1]. CMV establishes life-long latency in the host and can then be reactivated under immunocompromised states or cause a carrier-state [1, 4, 5]. CMV can affect immunocompromised hosts aggressively and is a significant cause of morbidity and mortality in solid organ transplant (SOT) recipients [1, 6]. SOT recipients are at highest risk in the first month after transplantation [6]. Serologic status of the donor, recipient, and the type of transplanted organ play a key role, as CMV disease is more likely in SOT recipients with no preexisting CMV-specific immunity [1, 6].

Clinical manifestations of CMV infection in SOT recipients include fever, malaise, leukopenia, and thrombocytopenia. CMV can also cause end-organ damage involving kidneys, pancreas, colon, lung, and CNS disease [1, 6]. Cutaneous CMV involvement is rare but when present can be rapidly fatal if not diagnosed quickly [7–10]. CMV cutaneous lesions can precede systemic disease. Thus, a high index of suspicion and early biopsy is needed for timely management to decrease morbidity and mortality [11]. Gastrointestinal (GI) tract CMV infections can cause colonic ulcerations, bleeding, and perforations with colitis and esophagitis as the two most common manifestations [12, 13]. We describe two atypical cases of CMV infection in immunocompromised hosts following successful organ transplantation.

## 2. Case 1: CMV Skin Lesion

**Figure 1:**
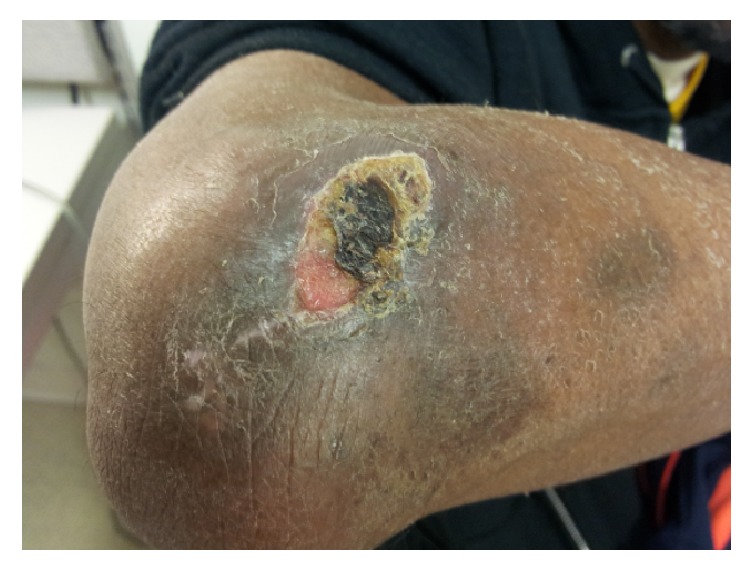
Cutaneous CMV ulceration of right elbow.

A 47-year-old male presented with a two-week history of back pain, generalized muscle aches, nausea, vomiting, diarrhea, and pain in his right elbow (Figure 1). Medical history included hypertension, hyperlipidemia, end-stage renal disease, renal transplant five years earlier, and pancreas transplant one year earlier. Medications included mycophenolic acid and tacrolimus for immunosuppression. Patient had not recently been on corticosteroids and had adequate allograft function with a baseline serum creatinine range of 1.7–1.9 mg/dL. Pretransplant studies revealed negative CMV IgG in the recipient and positive serology in the donor, but no additional risk factors for CMV infection were present. Patient was on trimethoprim-sulfamethoxazole for prophylaxis for the past 12 months and CMV prophylaxis with valganciclovir was taken for 6 months after transplantation and discontinued 6 months before presentation.

At presentation, the patient was febrile to 38 degrees Celsius. Physical exam was remarkable for tenderness in right costovertebral region and a tender, edematous, and erythematous scab on the right elbow with minimal drainage present. Labs revealed leukopenia with total white blood cell count of 3,000 mm^3^, hemoglobin of 10.2 g/dL, platelets of 184,000 mm^3^, and normal transaminases. Electrolytes were unremarkable with creatinine at baseline (1.9 mg/dL). Chest imaging was normal. He was admitted to the hospital and received empiric antimicrobials for a possible bacterial cellulitis.

**Figure 2:**
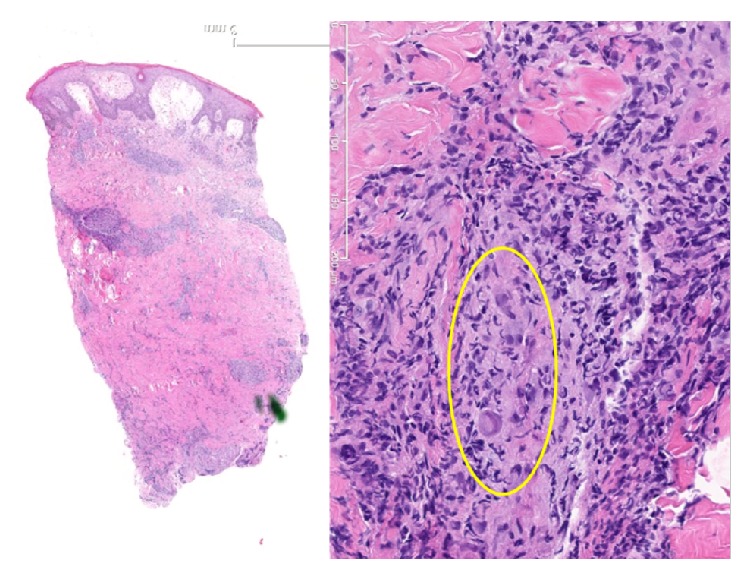
Histocytology stain showing CMV inclusion bodies from elbow biopsy.

His condition did not improve despite broad antibacterial coverage. A punch biopsy was performed on the lesion. The tissue was found to have multiple viral cytopathic inclusion bodies and a lymphocytic vasculitis concerning CMV infection. Further histopathologic studies confirmed the diagnosis (Figure 2). Serum PCR for CMV was reported at 21,000 copies. The patient was started on valganciclovir 900 mg every 12 hours. Antibacterials were stopped and patient was eventually discharged on a three-week course. The patient tolerated therapy and the lesions resolved.

## 3. Case 2: CMV Esophageal Lesion

A 50-year-old male presented with a one-day history of shortness of breath. Medical history included peripheral vascular disease, hyperlipidemia, diabetes, hypertension, end-stage renal disease, and cadaveric renal transplant 11 days prior to presentation. His immediate postoperative course was complicated by acute tubular necrosis and delayed graft function. The patient received IV methylprednisolone on the day of surgery followed by a 7-day prednisone taper. He received basiliximab and was subsequently switched to anti-thymocyte globulin (ATG) for induction therapy. Pretransplant studies revealed negative CMV IgG in the recipient and positive serology in the donor. He was started on valganciclovir and trimethoprim-sulfamethoxazole for prophylaxis on postoperative day 0, along with tacrolimus and mycophenolic acid for immunosuppression. He required two sessions of hemodialysis postoperatively. Allograft function recovered, with serum creatinine improving from 9.3 to 7.2 mg/dL by postoperative day 5. He was discharged home on day 7.

**Figure 3:**
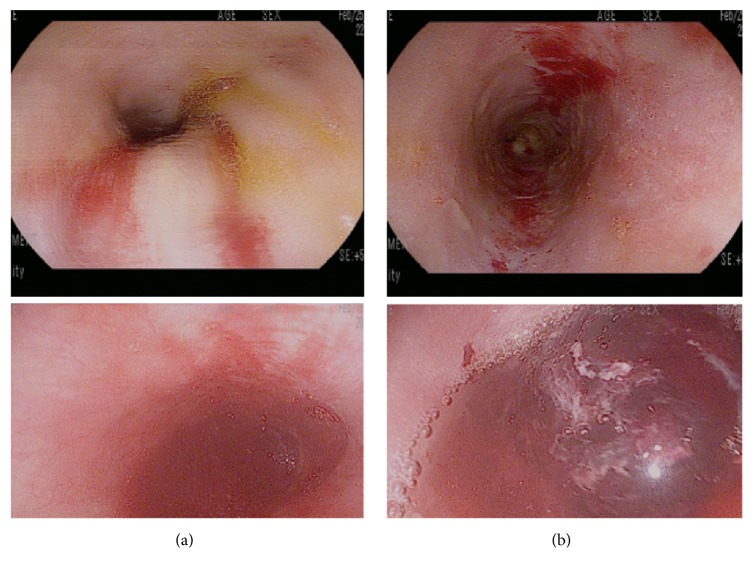
Esophagogastroduodenoscopy showing (a) duodenal ulcerations with adherent clots and (b) blackened middle and lower esophagus.

**Figure 4:**
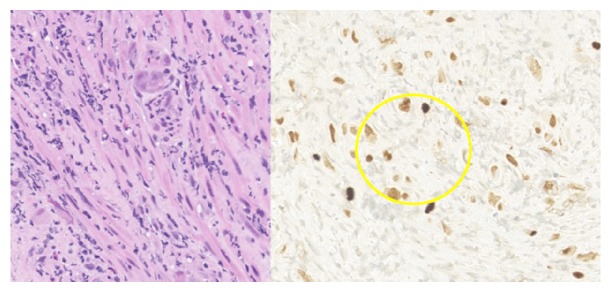
Histocytology stain indicating CMV inclusion bodies from esophageal biopsy.

At presentation, the patient developed respiratory failure and cardiopulmonary arrest. Return of spontaneous circulation was achieved after five minutes of cardiopulmonary resuscitation. Laboratory values were remarkable for white blood cell count of 16.5 mm^3^, platelet count of 166,000 mm^3^, and a hemoglobin level of 6.8 g/dL. Serum creatinine was 4.0 mg/dL. Liver function tests were normal. Chest imaging and renal ultrasound revealed no abnormalities. The patient was extubated five days later. He developed epigastric discomfort. Esophagogastroduodenoscopic (EGD) examination showed duodenal ulcerations with adherent clots and a blackened middle and lower esophagus thought to be secondary to ischemia (Figure 3). Serum CMV PCR detected no viral copies. Biopsies revealed viral inclusions throughout these lesions consistent with CMV (Figure 4). The patient was started on ganciclovir and clinically improved. He was transitioned to PO valganciclovir after one week and treated for a total of 21 days. Valganciclovir was subsequently changed to prophylactic doses and continued for 12 months.

## 4. Discussion

CMV infections have been recognized as a significant cause of morbidity and mortality in immunocompromised patients such as in solid organ transplant patients [7]. Skin ulcerations in immunocompromised patients merit a broad differential diagnosis including infectious and noninfectious causes. CMV involvement of skin has been described in the literature, but this condition remains rare. To our knowledge, there are fewer than 200 cases reported in the medical literature in the English language. Skin manifestations can include petechiae, nodules, ulcers, erosions, purpura, morbilliform eruptions, vesiculobullous lesions, papules, and verrucous lesions [9, 14–19]. There does not seem to be a predilection for the location of these lesions. The anogenital, perineum, heel, scrotum, axilla, or penile regions have all been reported to be involved [12, 20–22]. Painful ulceration of the tongue with CMV infection in renal transplant patients has also been reported [12]. Though these skin manifestations are heterogeneous, they all exhibit a typical histological appearance that includes vascular endothelial cytopathic changes and intranuclear inclusion bodies [11, 23]. Prior to advent of antiviral therapy, cutaneous CMV disease was associated with high mortality and was often diagnosed postmortem [23]. Early biopsy is imperative for reaching the diagnosis without delay, initiating appropriate therapy, and preventing further morbidity or mortality.

Gastrointestinal CMV disease is common in the first 6–12 months after transplantation and causes significant morbidity and mortality [24, 25]. The GI tract is the most common site of tissue-invasive CMV infection with colitis as the first most common site and esophagitis as the second most common [12, 13]. Failure to identify early stage CMV infection can cause further viremia, end-organ damage at other sites, and further damage to the GI microvasculature causing ulcerations, bleeding, and perforation [12]. Typical endoscopic findings of tissue-invasive CMV of the GI tract include shallow, erythematous erosions or localized ulcers [25]. Histopathology may show CMV inclusion bodies. Serum CMV PCR studies may yield negative results in patients with tissue-invasive disease; thus early biopsy for suspected tissue-invasive disease is recommended [1].

In the second case study, the patient presented 10 days after transplantation and had received ATG therapy as part of his induction therapy. ATG therapy increases the risk of CMV disease and is considered an independent risk factor [26, 27]. This likely contributed to his severe tissue-invasive disease. EGD revealed classic duodenal and esophageal ulcerations and immunohistochemical stains of the biopsy specimens revealed viral inclusion bodies consistent with CMV. Other rare gastrointestinal presentations of CMV infections include GI obstruction, perforation, thrombosis of large venous structures, splenic artery thrombosis, and pancreatitis [12]. Renal-allograft ureteral strictures have also been described [12].

We herein describe two rare manifestations of CMV disease in SOT transplant recipients. Despite being a well-recognized cause of morbidity and mortality in SOT recipients, CMV infection can present in atypical forms and pose a diagnostic challenge. High index of suspicion and early tissue biopsy are key for proper diagnosis and treatment.

## References

[1] Razonable R. R., Humar A. (2013). Cytomegalovirus in solid organ transplantation. *American Journal of Transplantation*.

[2] Cannon M. J., Schmid D. S., Hyde T. B. (2010). Review of cytomegalovirus seroprevalence and demographic characteristics associated with infection. *Reviews in Medical Virology*.

[3] Staras S. A. S., Dollard S. C., Radford K. W., Flanders W. D., Pass R. F., Cannon M. J. (2006). Seroprevalence of cytomegalovirus infection in the United States, 1988–1994. *Clinical Infectious Diseases*.

[4] Manuel O., Pang X. L., Humar A., Kumar D., Doucette K., Preiksaitis J. K. (2009). An assessment of donor-to-recipient transmission patterns of human cytomegalovirus by analysis of viral genomic variants. *Journal of Infectious Diseases*.

[5] Razonable R. R. (2005). Epidemiology of cytomegalovirus disease in solid organ and hematopoietic stem cell transplant recipients. *American Journal of Health-System Pharmacy*.

[6] Trimarchi H., Casas G., Jordan R. (2001). Cytomegalovirus maculopapular eruption in a kidney transplant patient. *Transplant Infectious Disease*.

[7] Colsky A. S., Jegasothy S. M., Leonardi C., Kirsner R. S., Kerdel F. A. (1998). Diagnosis and treatment of a case of cutaneous cytomegalovirus infection with a dramatic clinical presentation. *Journal of the American Academy of Dermatology*.

[8] Swanson S., Feldman P. S. (1987). Cytomegalovirus infection initially diagnosed by skin biopsy. *American Journal of Clinical Pathology*.

[9] Evans D. J., Williams E. D. (1968). Cytomegalic inclusion disease in the adult. *Journal of Clinical Pathology*.

[10] Toome B. K., Bowers K. E., Scott G. A. (1991). Diagnosis of cutaneous cytomegalovirus infection: a review and report of a case. *Journal of the American Academy of Dermatology*.

[11] Pariser R. J. (1983). Histologically specific skin lesions in disseminated cytomegalovirus infection. *Journal of the American Academy of Dermatology*.

[12] Ardalan M. (2012). Rare presentations of cytomegalovirus infection in renal allograft recipients. *Nephro-Urology Monthly*.

[13] Kotton C. N., Kumar D., Caliendo A. M. (2013). Updated international consensus guidelines on the management of cytomegalovirus in solid-organ transplantation. *Transplantation*.

[14] Penneys N. S., Hicks B. (1985). Unusual cutaneous lesions associated with acquired immunodeficiency syndrome. *Journal of the American Academy of Dermatology*.

[15] Feldman P. S., Walker A. N., Baker R. (1982). Cutaneous lesions heralding disseminated cytomegalovirus infection. *Journal of the American Academy of Dermatology*.

[16] Robson G., Mackay I. (1969). Generalized cytomegalovirus infection in a patient with lupoid hepatitis. *Australasian Annals of Medicine*.

[17] Minars N., Silverman J. F., Escobar M. R., Martinez A. J. (1977). Fatal cytomegalic inclusion disease: associated skin manifestations in a renal transplant patient. *Archives of Dermatology*.

[18] Meyers J. D., Flournoy N., Thomas E. D. (1980). Cytomegalovirus infection and specific cell-mediated immunity after marrow transplant. *Journal of Infectious Diseases*.

[19] Guo R. F., Gebreab F. H., Tang E. H., Piao Z., Lee S. S., Perez M. L. (2015). Cutaneous ulcer as leading symptom of systemic cytomegalovirus infection. *Case Reports in Infectious Diseases*.

[20] Lambert E. M., Strasswimmer J., Lazova R., Antaya R. J. (2004). Cytomegalovirus ulcer: successful treatment with valganciclovir. *Archives of Dermatology*.

[21] Drago F., Aragone M. G., Lugani C., Rebora A. (2000). Cytomegalovirus infection in normal and immunocompromised humans: a review. *Dermatology*.

[22] Prasad N., Jain M., Gupta A., Sharma R. K., Agarwal V. (2010). An unusual case of CMV cutaneous ulcers in a renal transplant recipient and review of literature. *NDT Plus*.

[23] Golden M. P., Hammer S. M., Wanke C. A., Albrecht M. A. (1994). Cytomegalovirus vasculitis. Case reports and review of the literature. *Medicine*.

[24] Silva M., Silva R., Macedo G. (2016). Extensive esophageal ulceration in a renal transplant patient. *GE Portuguese Journal of Gastroenterology*.

[25] Helderman J. H., Goral S. (2002). Gastrointestinal complications of transplant immunosuppression. *Journal of the American Society of Nephrology*.

[26] Taherimahmoudi M., Ahmadi H., Baradaran N. (2009). Cytomegalovirus infection and disease following renal transplantation: preliminary report of incidence and potential risk factors. *Transplantation Proceedings*.

[27] Low C. Y., Hosseini-Moghaddam S. M., Rotstein C., Renner E. L., Husain S. (2017). The effect of different immunoprophylaxis regimens on post-transplant cytomegalovirus (CMV) infection in CMV-seropositive liver transplant recipients. *Transplant Infectious Disease*.

